# Gamma-Delta T-Cell Lymphoma Following Allogeneic Stem Cell Transplant for Primary Myelofibrosis

**DOI:** 10.7759/cureus.10301

**Published:** 2020-09-07

**Authors:** Robert Hoard, George Shahin, Florin D Andreca, Michael Osswald

**Affiliations:** 1 Internal Medicine, San Antonio Uniformed Services Health Education Consortium (SAUSHEC), San Antonio, USA; 2 Internal Medicine: Hematology / Oncology, San Antonio Uniformed Services Health Education Consortium (SAUSHEC), San Antonio, USA; 3 Hematology / Oncology, Wright-Patterson Air Force Base (WPAFB), Dayton, USA; 4 Hematology / Oncology, Brooke Army Medical Center, San Antonio, USA

**Keywords:** gamma-delta t-cell, lymohoma, myelofibrosis

## Abstract

Primary myelofibrosis (PMF) is a disease that affects the bone marrow. It presents with cytopenias, hepatospleomegaly accompanied with extramedullary hematopoiesis, and often with constitutional symptoms. Cytotoxic gamma-delta T-cells are considered a distinct hepatosplenic lymohoma. This is a case presentation of a 43-year-old male with an initial diagnosis of PMF who underwent allogeneic stem cell transplantation complicated by primary graft failure. He subsequently underwent a partial splenic embolization; however, he quickly developed a fulminant hepatosplenic gamma-delta T-cell lymphoma which led to his death that was diagnosed posthumously. PMF has been known to transform into an acute myeloid leukemia, but there has been no established link with gamma-delta T-cell lymphoma.

## Introduction

Primary myelofibrosis (PMF) is a rare but aggressive myeloproliferative neoplasm (MPN) that often kills by transformation to acute myeloid leukemia. The only current curative treatment is an allogeneic stem cell transplant. Peripheral T-cell lymphomas comprise a separate heterogeneous group of neoplasms, including hepatosplenic T-cell lymphoma (HSTL). The gamma-delta subgroup of HSTL has an aggressive clinical course with poor prognosis and an unknown pathogenesis, though chronic immunosuppression in solid organ and hematopoietic transplants and prolonged antigenic exposure are proposed risk factors. HSTL is a group of post-thymic, mature lymphoid malignancies, and they represent approximately 10%-15% of all non-Hodgkin's lymphomas (NHLs). T-cells naturally play a role in the innate, nonspecific immune response. They do not express the histocompatibility complex restrictions and can be divided into two subpopulations: Vdelta1, mostly represented in the intestine, and Vdelta2, prevalently located in the skin, tonsils, and lymph nodes. Two entities are recognized by the WHO classification: hepatosplenic gamma-delta T-cell lymphoma (HSGDTL) and primary cutaneous gamma-delta T-cell lymphoma (PCGDTL). Diagnosis of HSTL requires a high clinical suspicion given the subtlety of clinical manifestations that can be similar to myelofibrosis. Diagnosis requires invasive procedures like core needle biopsy of liver, bone marrow biopsy, or by examination of splenectomy specimens [1-10].

## Case presentation

A 43-year-old male initially presented in October 2016 with several weeks of abdominal pain, drenching sweats, recurrent fevers, and early satiety. During the initial evaluation, exam was notable for palpable splenomegaly and labs demonstrated pancytopenia. A bone marrow biopsy revealed a markedly hypercellular marrow (90%-95%) with erythroid hyperplasia; dysplasia in both the erythroid and megakaryocytic lines; absent stainable iron stores; and fewer than 2% blasts. Fluorescence in situ hybridization (FISH) panel revealed a JAK2 V617F mutation and was negative for BCR-ABL, suggesting a diagnosis of MPN/ PMF overlap by 2016 WHO criteria. He was started on ruxolitinib with significant improvement in his constitutional symptoms and reduction in splenomegaly.

In March 2017, he was evaluated for an allogeneic stem cell transplant. At that time, he was still asymptomatic with a nonpalpable spleen. Given his lack of symptoms and response to ruxolitinib, the decision was made to postpone transplantation. His disease continued to be well controlled until November 2017 when his splenomegaly, constitutional symptoms, and pancytopenia recurred. Repeat bone marrow biopsy showed no increase in CD34+ blasts or abnormal T-cell or B-cell population, a cellularity more than 95% and with the myeloid:erythroid ratio of 8:1. The reticulin stain showed mild fibrosis with no overt dysplasia. A relative increase in natural killer (NK) cells at 8.8% was noted. Cytogenetics and FISH panels were normal. Overall, the findings were considered to be compatible with the clinical history of treated PMF. He was classified as high risk with five points based on having constitutional symptoms, hemoglobin <10 g/dL, circulating blasts >1%, and platelets <100 x 109 /L when calculated with the Dynamic International Prognostic Scoring System Plus (DIPSS-Plus). Stem cell transplant was determined to be the most appropriate next step in treatment.

**Figure 1:**
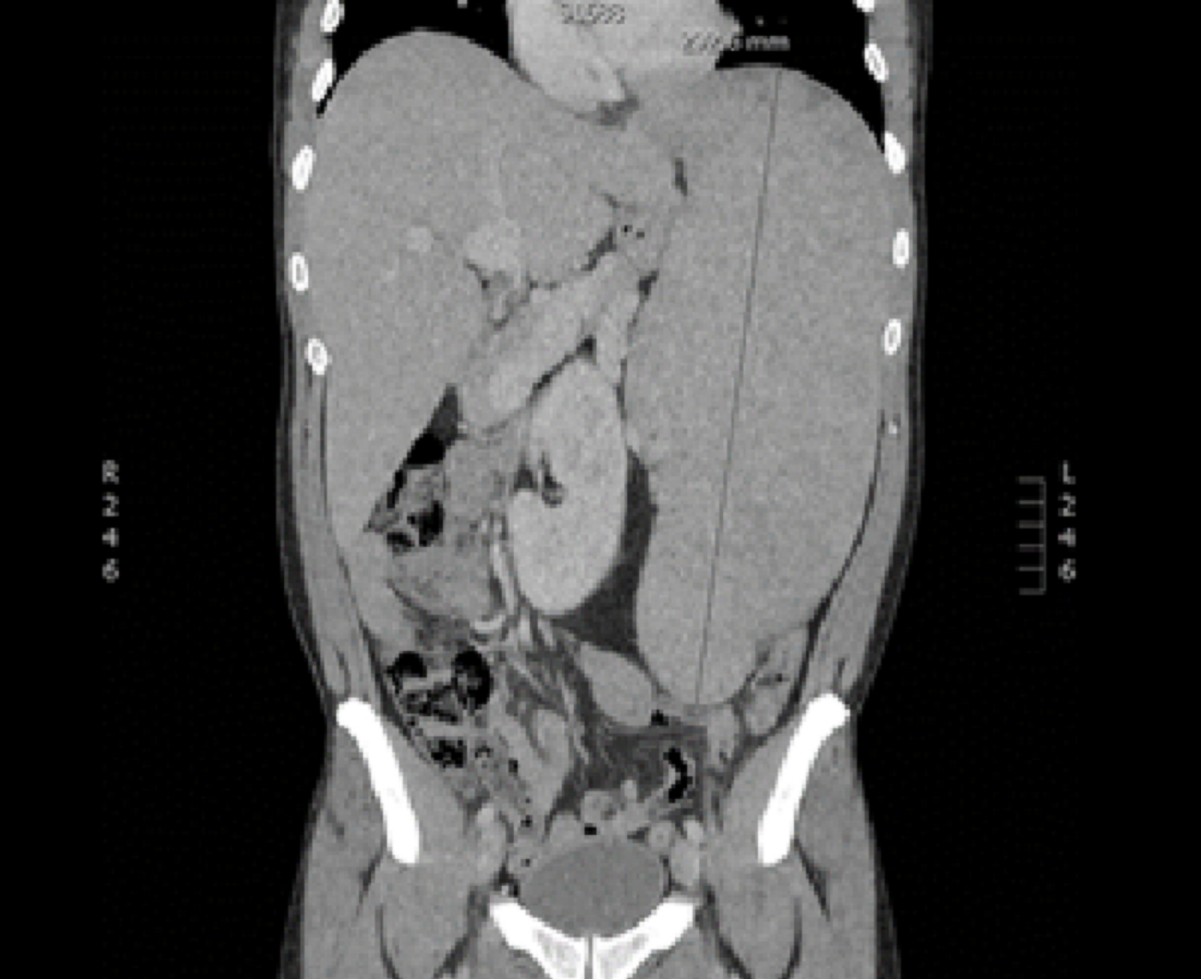
Pretransplant splenomegaly.

During the transplant workup and donor search, the patient was initiated on lenalidomide and prednisone; however, treatment was complicated by cytopenias requiring dose reductions. Ultimately the decision was made for cessation of therapy. Pretransplant imaging demonstrated a spleen size of 29 cm (Figure 1). In April 2018 after extensive search no matched related or unrelated donor was found. A stem cell donor was identified and he received an allogeneic stem cell transplant on using a reduced-intensity conditioning regimen with cyclophosphamide, fludarabine, and total body irradiation with a 9/10 mismatched unrelated donor. Below is the patient’s MRI indicating pretransplant splenomegaly (Figure 1).

**Figure 2:**
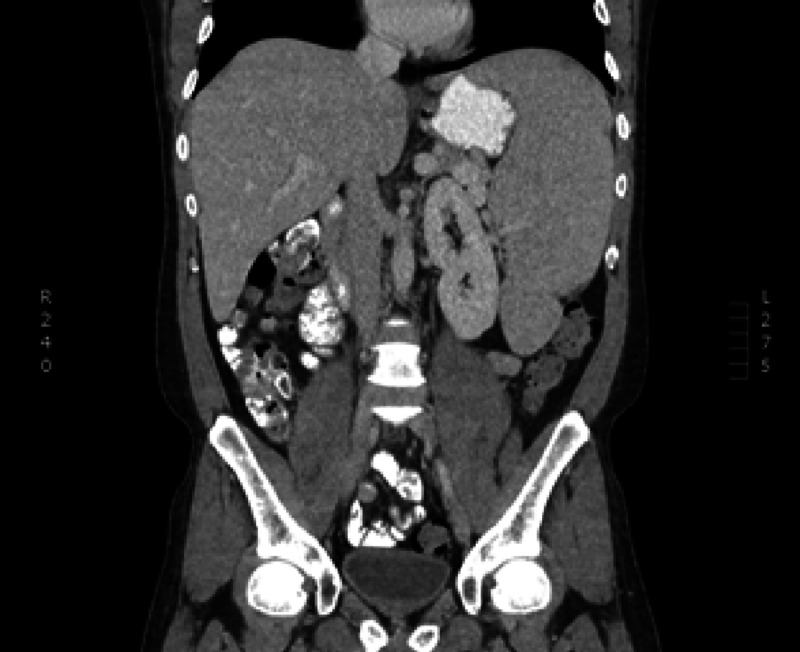
Post-transplant splenomegaly.

The patient was treated with applicable graft-versus-host-disease tacrolimus and mycophenolate mofetil post-transplant with addition of posaconazole, valacyclovir, and trimethoprim/sulfamethoxazole. At day 28 chimerisms showed 4% donor cells with engrafted neutrophils around day +25 and platelets on day +36, but he had not yet engrafted. A repeat chimerisms on day 36 showed 0% donor cells engrafted, consistent with graft failure with autologous recovery. The allogenic stem cell transplant or the chemotherapy did result in resolution of constitutional symptoms and significant improvement in symptoms and splenomegaly as seen in the post-transplant image (Figure 2).

Another bone marrow biopsy was performed after count recovery showed adequate hematopoiesis, with increased iron storages, and no ringed sideroblasts. There was focal mild reticulin fibrosis and no dysplasia with blast percentage of 1%. The overall findings were nonspecific, and there was no overt morphologic evidence of the previously diagnosed PMF. The cytogenetics showed a normal male karyotype with a negative FISH for acute myeloid leukemia or myelodysplastic syndrome panel. Flow cytometry showed no monoclonal B-cell or abnormal T-cell population, but the NK cells were increased to 15% of the total lymphocytes. Approximately four months after his transplant, his index symptoms returned.

**Figure 3:**
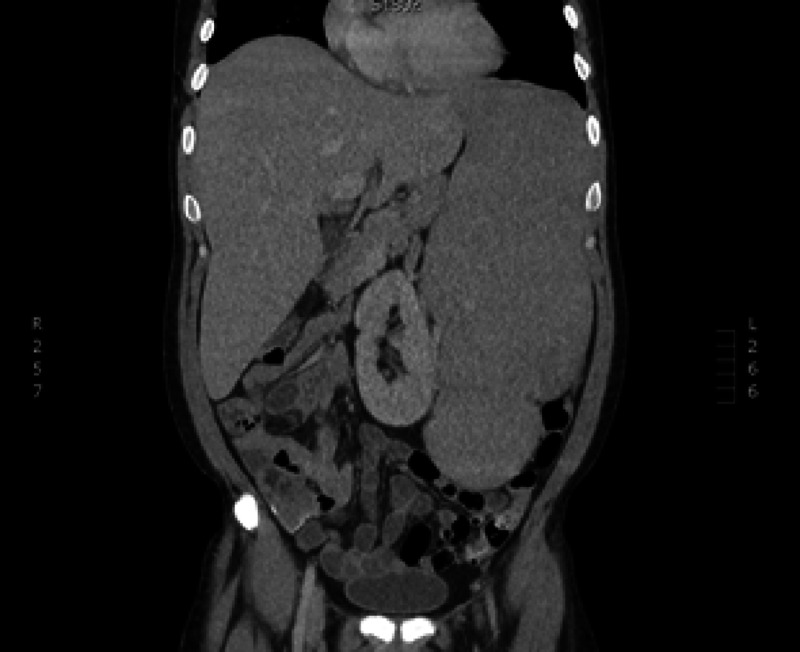
Relapsed splenomegaly.

Relapsed splenomegaly at four months post-transplant (Figure 3).

The patient was to be referred to a high-volume transplant center with a capability to reduce alloantibodies against his original donor; however, he developed rapid progressive cytopenias and symptoms that precluded travel. Splenectomy was discussed as a temporizing measure; however, the surgical procedure was deemed too high risk in a setting of severe thrombocytopenia refractory to transfusions. He was referred to radiation oncology and started splenic radiation therapy. Eventually, the radiation treatment was discontinued due to progressive neutropenia. He then underwent a partial splenic embolization designed to target the lower half of the spleen to avoid complication from postembolization syndrome.

**Figure 4:**
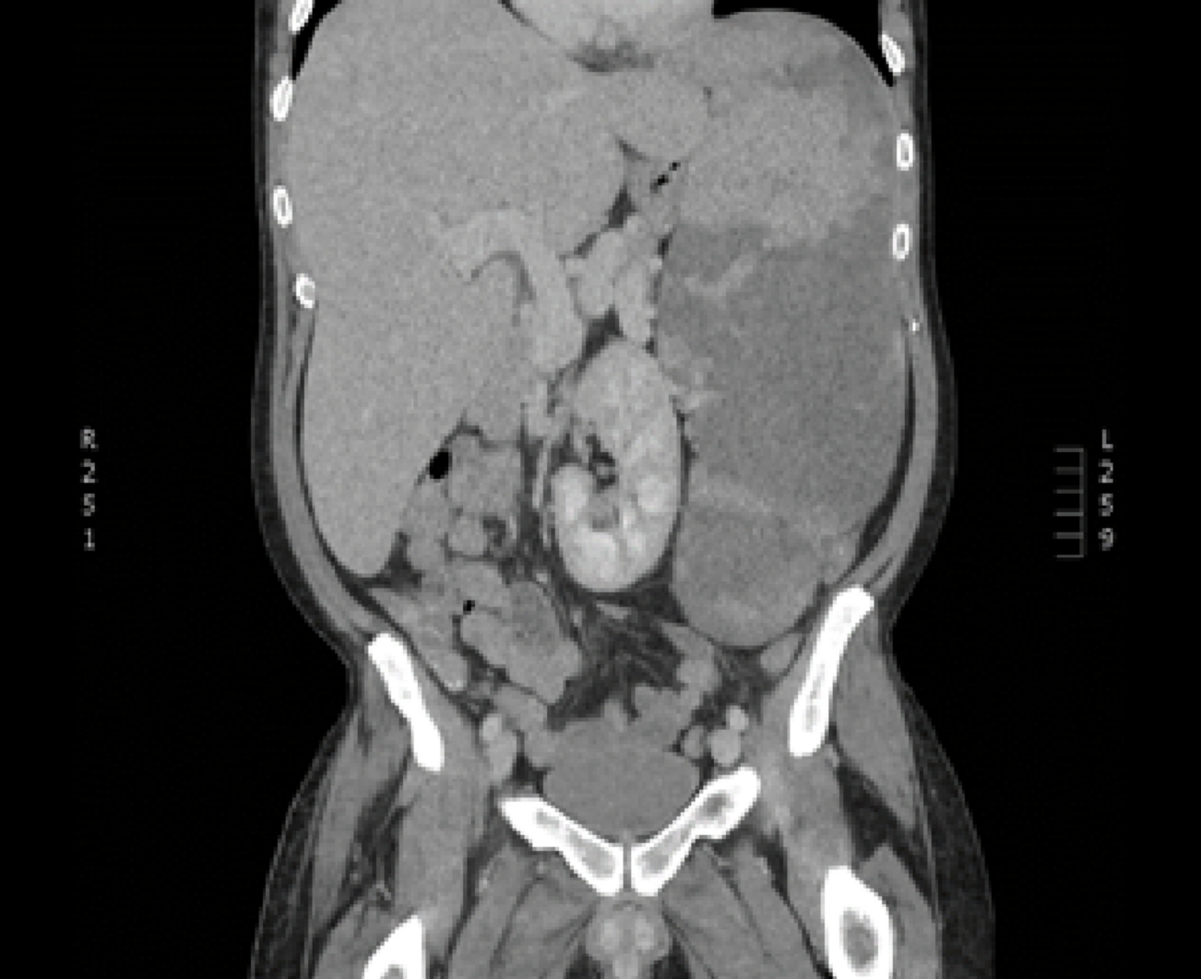
Postembolization spleen.

Below is an MRI picture of the postembolization spleen (Figure 4).

**Figure 5:**
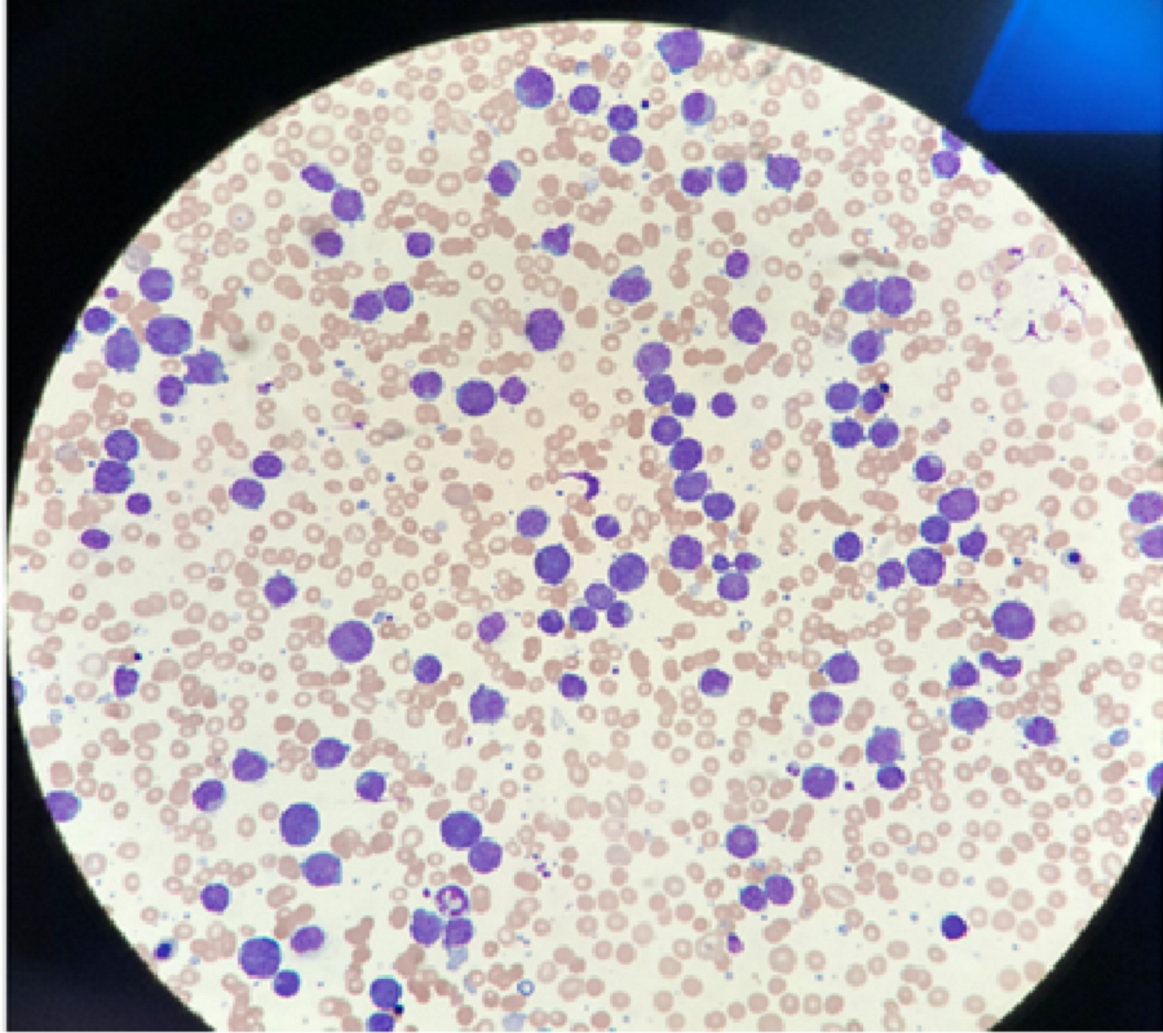
Peripheral smear.

Two weeks postprocedure, the patient presented with a severe deterioration in his status, multi-organ failure, and an exponential rise in his white blood cell (WBC) from 4000 to 240,000 (x10^3) over a 24-hour period, which eventually led to the patient’s death. Below there is an image of the peripheral smear showing a mononuclear lymphoid cell population just prior to his death (Figure 5).

**Figure 6:**
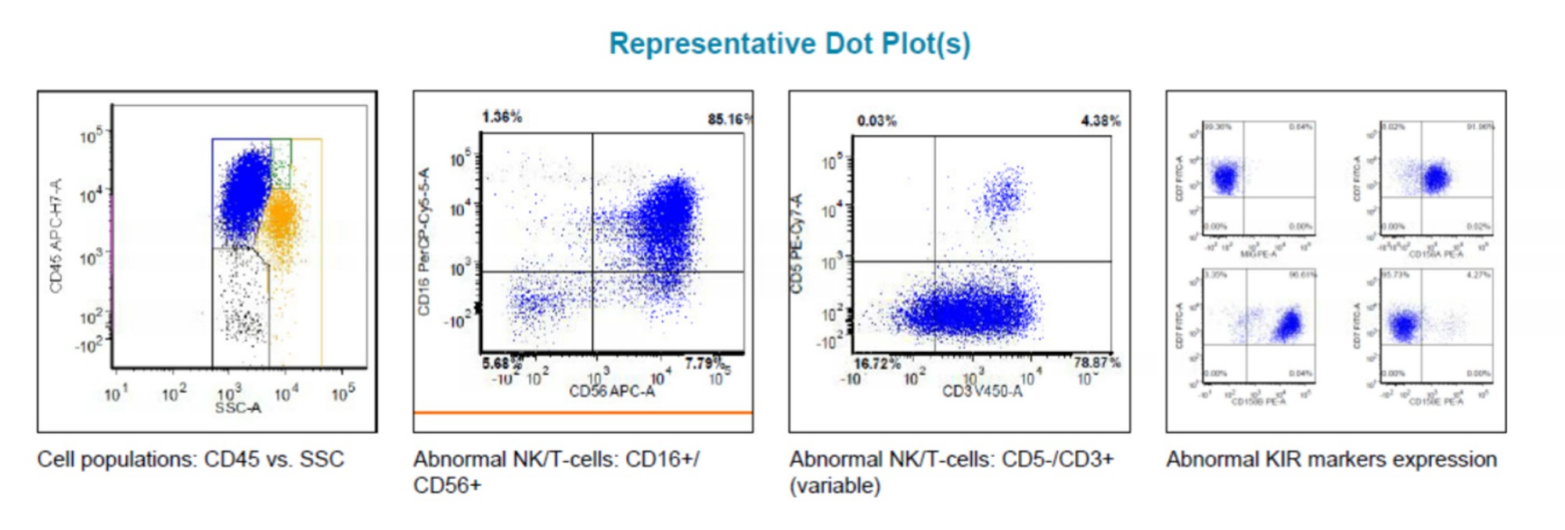
Peripheral blood flow cytometry results.

The flow cytometry of the peripheral blood showed the abnormal T-cell population accounting for 85% of the gated events and expressing CD3 and CD56 positivity. T-cell receptor gene rearrangement studies demonstrated clonal abnormalities. In combination with flow cytometry studies suggesting an NK cell phenotype (CD 2+, CD 7+, CD 5-, CD 56 + and CD 16 + with abnormal KIR markers expression), a gamma-delta T-cell lymphoma/leukemia was favored (Figure 6) [8-9].

## Discussion

A concurrent HSGDTL in the setting of PMF is rare, as it accounts for <1% of non-Hodgkin’s lymphoma (NHL). The natural behavior of HSGDTL is characterized by low response rates, poor treatment tolerability, common early progression of disease, and dismal survival.

The available literature on gamma-delta T-cell lymphomas mostly consists of case reports or small cumulative series. A standard treatment option for patients with HSGDTL has not been yet established because of the extreme rarity of this clinical entity. Our extensive literature review indicated only one other case discussing simultaneous HSTL and PMF. The case was published in 2014 April-June edition of the *Avicenna Journal Medicine* of a simultaneous HSGDTL and myelofobrosis (JAK2 negative). The results of JAK2 analysis in their case did not allow the authors to reach a final unequivocal decision whether or not the myelofibrosis was considered a primary or secondary etiology; as JAK2 mutation is only positive in 50% of PMF cases. We found no other cases of a simultaneous PMF and HSGDTL. Our case is unique from the Gabali et al. case report as our patient likely had a primary rather than a secondary myelofibrosis (due to HSTL) given the presence of JAK2 positive gene mutation [1-10]. 

Our patient developed a fulminant T-cell lymphoma consistent with HSTL, gamma-delta subtype, leading to rapid multi-organ failure and death. Firstly, whether a diagnosis of HSGDTL was present at the time of initial myelofibrosis diagnosis or developed as a result of chronic immunosuppression was undetermined. Secondly, the potential of spleen-directed therapy in relation to observed leukemogenesis is undetermined. Thirdly, whether splenic embolization should be utilized as a treatment of choice for patients with PMF and questionable additional underlying malignancy.

## Conclusions

This case report suggests that a T-cell lymphoma gamma-delta type can coexist with a PMF (confirmed JAK2 positive). More importantly, it also raises a flag to the clinician to maintain an open differential and reconsider additional malignancies when treating a refractory disease. This case serves to highlight the coexistence of two extremely rare neoplasms with phenotypic overlap making diagnosis difficult, but essential to delineate prognosis and treatment. This case highlights the importance of treating the underlying diagnosis. Perhaps if HSGDTL was known earlier, a different approach to spleen-directed therapy or earlier action for splenectomy would have been recommended. It is not known if the patient had indolent HSGDTL during his initial diagnosis. In addition it is unclear whether spleen-directed therapies somehow accelerated the expression of abnormal T-cell gamma delta cells. Further research is warranted to determine the effects of splenectomy, splenic radiation, or splenic embolization on malignant expression of T-cell lymphomas. Furthermore, continued research of the pathophysiology and treatment of gamma-delta T-cell lymphoma are warranted given this rare but insidious disease.
